# The Out-Patient Problem

**Published:** 1897-10-30

**Authors:** 


### The Institutional Workshop.

### THE OUT-PATIENT PROBLEM.
### A SOLUTION IN SIGHT.

It is with great satisfaction that we are able to announce that there is at last a prospect that the divergent views held on the subject of the reform of the out-patient departments of the hospitals, and especially of the Metropolitan hospitals, are in a fair way of being reconciled. Since the beginning of the present year the hospital authorities, the general practitioners through their new organisation the Hospital Reform Association, and the Distribution Committee of the Metropolitan Hospital Sunday Fund, have all been quietly working at the problem. They have gone over much of the old ground, have wisely eliminated irrelevant matter, and have so gradually brought the point at issue into focus. Each of the three bodies working entirely independently and without reference to each other have arrived at a decision, and it is of the greatest moment that that decision is practically identical.

The most important of the recommendations of the committee appointed by the conference of general hospitals is that each general hospital should have an enquiry officer or out-patient clerk to enquire into the means of the person applying for free treatment, the duties of such officer to include: (*a*) The prevention of abuse of the charity by persons able to pay for medical treatment. (*b*) The referring of patients already in receipt of parish relief and such as are destitute to the poor law authorities—a large proportion of those patients really needing proper food rather than medicine.

At the second meeting of the Hospital Reform Association last week it was stated from the chair that the committee appointed to consider what remedy can be applied to the evils attaching to the out-patient department as at present conducted would take steps to organise deputations of medical men to confer with the honorary medical staff of each of the more important hospitals, with a view to inducing them to use their influence to secure the immediate appointment of a registration officer or almoner to inquire into the means of the persons applying for free treatment in the out-patient departments. At a meeting of the Council of the Metropolitan Hospital Sunday Fund held at the Mansion House on Wednesday, the 27th inst., the Distribution Committee brought up a report in which they recommended that efforts should be made to induce each hospital to appoint efficient persons to make inquiries concerning the patients attending out-patient departments with the view of preventing any abuse. This report was adopted, and the Council unanimously resolved to recommend an addition to the laws of the constitution providing that any hospital employing such an officer shall be regarded by the Distribution Committee as having provided an important factor in determining its merits.

It will thus be seen that, as we have stated, the managers of the general hospitals, the Distribution Committee and the Council of the Hospital Sunday Fund, and the general practitioners of medicine, as represented by the Hospital Reform Association, are all agreed as to the particular step which it is desirable to take to secure the necessary reforms. This unanimity points to a practical solution of one of the most difficult of hospital questions, which has given rise to ceaseless controversy during the last 30 years.

Of course, the appointment of the registration officer will merely establish the necessary machinery by means of which it will be possible to provide that each out-patient admitted to treatment shall obtain relief on his merits, because due regard will be had to the social circumstances and to the medical fitness of each applicant. There remains the question of what step ought to be taken to secure the immediate admission of every indigent person whose medical needs entitle him to hospital treatment, whilst ruling out every one whose means are such as to enable him to procure necessary medical aid by payment apart from the hospitals. We have given a good deal of consideration to this question, and we have come to the conclusion that, if the hospitals would institute a rule that they would give no relief to any person who was in a position to subscribe upwards of 10s. a year to the hospitals, except in special circumstances where they were sent to the hospitals by their medical attendants for consultation, hospital abuse would be put an end to at once. We are further of opinion that the patients admitted, who are above the indigent class, would gladly welcome the opportunity of showing the value they attach to the hospitals and their desire to do their best to help to support them, if they were given facilities for subscribing small sums annually on a plan which would enable every subscriber to show exactly what he had done in this respect year by year. The Subscription Book and Stamp Album, which is issued by the Prince of Wales's Hospital Fund for London, with the object of enabling subscribers of small sums of one to ten shillings per annum to possess evidence of the fact that they are regular subscribers, through the Prince of Wales's Hospital Fund, to the metropolitan hospitals, gives the managers of these institutions the means of raising a considerable revenue from small annual subscribers, and is exactly what is wanted. This Subscription Book and Stamp Album will give to every person who frequents the hospitals an opportunity of subscribing according to their means a small sum each year to these institutions. If the hospital authorities will co-operate with the Prince of Wales's Fund, as we hope they may agree to do, the problem of raising the amount received in annual subscriptions by the Metropolitan hospitals to a sum at least equal to that received from the same source by provincial hospitals will in all probability be solved before the end of 1898. Assuming that the average annual subscription from the classes we refer to is but half-a-crown per annum, in the aggregate these half-crowns will amount to a sum so important as to place every hospital which adopts the system in a stronger financial position than its most enthusiastic supporters believe possible of accomplishment. This scheme for affording facilities to small annual subscribers to the hospitals is not open to the objection of the fee system, because it enables every person to give that sum per annum in the form of an annual subscription, which represents the ful giving power of each individual concerned. The more we encourage thrift among the classes who seek hospital relief, the more we shall build up their character, and the more we shall secure that they will hesitate to take hospital aid unless they are fully persuaded that they cannot obtain the medical relief which they require except at a hospital. If every Metropolitan hospital of importance consents to appoint a registration officer or almoner, the work of these officials will be much simplified and aided by the issue of the attractive Subscription Book and Stamp Album which the hospitals owe to the energy and administrative skill of those who are responsible for the administration of the Prince of Wales's Fund. We shall have more to say on this point on another occasion, but we hope that the members of the medical profession, as well as the hospital managers, will do their utmost to make the impending changes in the method of administering the out-patient department and the encouragement of small annual subscribers to the hospital an undoubted permanent success.

